# Sertoli Cell Number Defines and Predicts Germ and Leydig Cell Population Sizes in the Adult Mouse Testis

**DOI:** 10.1210/en.2017-00196

**Published:** 2017-07-05

**Authors:** Diane Rebourcet, Annalucia Darbey, Ana Monteiro, Ugo Soffientini, Yi Ting Tsai, Ian Handel, Jean-Luc Pitetti, Serge Nef, Lee B. Smith, Peter J. O’Shaughnessy

**Affiliations:** 1 Institute of Biodiversity, Animal Health and Comparative Medicine, University of Glasgow, Glasgow G61 1QH, United Kingdom; 2 Medical Research Council Centre for Reproductive Health, The Queen’s Medical Research Institute, University of Edinburgh, Edinburgh EH16 4TJ, United Kingdom; 3 The Roslin Institute and Royal (Dick) School of Veterinary Studies, University of Edinburgh, Midlothian EH25 9RG, United Kingdom; 4 Department of Genetic Medicine and Development, University of Geneva, 1211 Geneva 4, Switzerland; 5 Faculty of Science, University of Newcastle, Callaghan, New South Wales 2308, Australia

## Abstract

Sertoli cells regulate differentiation and development of the testis and are essential for maintaining adult testis function. To model the effects of dysregulating Sertoli cell number during development or aging, we have used acute diphtheria toxin−mediated cell ablation to reduce Sertoli cell population size. Results show that the size of the Sertoli cell population that forms during development determines the number of germ cells and Leydig cells that will be present in the adult testis. Similarly, the number of germ cells and Leydig cells that can be maintained in the adult depends directly on the size of the adult Sertoli cell population. Finally, we have used linear modeling to generate predictive models of testis cell composition during development and in the adult based on the size of the Sertoli cell population. This study shows that at all ages the size of the Sertoli cell population is predictive of resulting testicular cell composition. A reduction in Sertoli cell number/proliferation at any age will therefore lead to a proportional decrease in germ cell and Leydig cell numbers, with likely consequential effects on fertility and health.

## Teaser

Sertoli cell knockout studies show that the numbers of Leydig cells and germ cells that develop and are maintained in the testis are directly proportional to the number of Sertoli cells.

Development and function of the testes require a complex orchestration of cell differentiation, proliferation, and communication in both fetal and postnatal life. Initiation of this cascade is dependent on the action of Sertoli cells. These cells form in the coelomic epithelium (1) and go on to induce formation of the seminiferous tubules and subsequent development of the fetal Leydig cell population (2). The number of Sertoli cells increases exponentially during fetal life in mice and humans and then slows after birth, reaching adult levels by early puberty (3–5).

Recent cell ablation studies from our group have shown that during the proliferation phase, Sertoli cells continue to regulate critical aspects of testis development (6–9). When Sertoli cells are totally ablated in the neonate, for example, tubule structure is lost, the peritubular myoid cells dedifferentiate, and subsequent differentiation and development of the adult population of Leydig cells is severely restricted (9). In the adult, Sertoli cells are essential for maintenance of spermatogenesis, and ablation of the Sertoli cells in the adult is associated with loss of germ cells (9). More surprisingly, however, Sertoli cell ablation in the adult also leads to loss of 70% of the adult Leydig cell population (8). Sertoli cells therefore act as central regulators of both testis development and adult function, so any developmental dysregulation that impacts Sertoli cell numbers, in either fetal or postnatal life, could have notable knock-on effects in other cell types. This would be likely to affect overall testis function in adulthood and exacerbate the effects of aging on reproduction and overall health (10–15).

Ablation of a total cell population is a very powerful technique for looking at control mechanisms in a tissue (7). With respect to normal development/aging and likely defects, however, complete loss of one cell type is very unlikely. More feasibly, reductions in cell numbers may be expected from a change in proliferation rates or an increase in apoptosis induced, for example, through exposure to toxicants *in utero* or by aging. The mouse diphtheria toxin (DTX) model of Sertoli cell ablation described recently (8, 9) provides a unique opportunity to examine the impact of reducing the size of the Sertoli cell population by defined amounts at different stages of development. The major advantages of this system are that it is acute (*i.e.,* Sertoli cell death occurs within 24 hours) and that it is specific to Sertoli cells (*i.e.,* no off-target effects on other cell types) (8, 9). It is an advance over previous correlative studies, because it allows cause-and-effect relationships to be defined without relying on specific gene knockout or endocrine modulation (with potential confounder effects) to alter Sertoli cell numbers.

Using this approach, we demonstrate that the size of the Sertoli cell population that forms during development regulates and maintains the overall cellular composition of the adult testis, defining numbers of germ cells and also numbers of Leydig cells present in the adult testis. We have also used these data to train age-category−stratified linear models of Sertoli cell numbers, which we show can be used as predictive biomarkers of overall testicular cell composition in development and in adulthood. Together, these findings demonstrate that Sertoli cells, and particularly Sertoli cell number, are key therapeutic targets in efforts to modulate adult testicular cell populations and functions in support of lifelong male health.

## Materials and Methods

### Animals and treatments

All animal studies passed local ethical review and were conducted with licensed permission under the UK Animal Scientific Procedures Act (1986), Home Office License No. PPL 70/8804. Mice with Sertoli cell−specific induction of the DTX receptor (iDTR mice) or Sertoli cell−specific induction of the DTX A-chain (DTA mice) were generated on mixed backgrounds as previously described (9). In both groups of mice, expression of the transgene was under control of anti-Müllerian hormone (AMH) promoter–Cre; thus, in DTA mice, Sertoli cell ablation will occur shortly after first expression of AMH, which occurs at about embryonic day (e) 13.5 (16) (Supplemental Fig. 1). Neonatal (2 days) or adult (50 days) male iDTR mice were treated with a single subcutaneous injection of DTX (0.01 to 100 ng) to induce Sertoli cell ablation and were euthanized up to 90 days after treatment (Supplemental Fig. 1). Serum was collected for measurement of luteinizing hormone (LH) and testosterone levels in each animal.

### Stereology

Testes were fixed for 6 hours in Bouin solution and then stored in 70% ethanol before being embedded in Technovit 7100 resin (Heraeus Kulzer GmbH, Hanau, Germany), cut into sections (20 μm), and stained with Harris hematoxylin and eosin. Total testis volume was estimated using the Cavalieri principle (17), and the optical disector technique (18) was used to count the number of Leydig cells, Sertoli cells, and germ cells in each testis. The numerical density of each cell type was estimated using an Olympus BX50 microscope fitted with a motorized stage (Prior Scientific Instruments, Cambridge, UK) and Stereologer software (Systems Planning Analysis, Alexandria, VA). Each cell type was recognized by its position and morphology as described previously (8, 19). Mean Leydig cell volume in each sample was measured using the uniform isotropic random rotator method (20) available within the Stereologer software. Eight lines per cell profile were measured, and between 150 and 200 cells were sampled per testis. To illustrate testis histology, semithin 2.5-µm sections were cut from tissues embedded in resin and stained with hematoxylin and eosin.

### Immunohistochemistry

Tissues were fixed in Bouin solution for 6 hours, stored in 70% ethanol, and embedded in paraffin. Sections (5 µm) were dewaxed in xylene and rehydrated, and antigens were retrieved in a pressure cooker with 0.01 M of citrate buffer (pH 6.0). To quench endogenous peroxidase activity, slides were incubated in 0.3% hydrogen peroxide (volume-to-volume ratio) in Tris-buffered saline for 30 minutes at room temperature. Nonspecific activity was blocked using the appropriate normal blocking serum for 30 minutes followed by overnight incubation at 4°C with the primary antibody diluted in blocking serum. The primary and secondary antibodies used in this study are detailed in Supplemental Table 1. After washing, slides were incubated for 30 minutes at room temperature with the appropriate secondary antibody. For immunofluorescence, peroxidase-conjugated secondary antibody was used diluted 1/200 in blocking serum. Sections were incubated for 10 minutes at room temperature with the fluorescein Tyramide Signal Amplification system (TSA™; PerkinElmer) diluted 1/50 according to the manufacturer’s instructions. Sections were then counterstained in Sytox Green (Molecular Probes; Life Technologies, Paisley, UK) for 10 minutes at room temperature and mounted in PermaFluor mounting medium (Thermo Fisher Scientific, Renfrew, UK). Slides were scanned using an LSM 710 confocal microscope and ZEN 2009 software (Carl Zeiss Ltd, Hertfordshire, UK). Sections incubated with no primary antibody were used as negative controls. To check reproducibility, sections from at least three different animals in each group were tested, and sections from vehicle- and DTX-treated animals were processed simultaneously on the same slide.

### RNA extraction, reverse transcription, and real-time polymerase chain reaction

Levels of specific messenger RNA transcripts were measured by real-time polymerase chain reaction (PCR). Total RNA was extracted from whole testes of each animal using TRIzol (Life Technologies, Paisley, UK). At the start of the extraction process, 5 ng external standard RNA (luciferase) was added to each sample for normalization of data (21). Reverse transcription, primer design, and real-time PCR were carried out as previously described (22, 23). The real-time PCR studies were carried out at an annealing temperature of 63°C, extension at 72°C, and denaturation at 95°C for 40 cycles. Dissociation curves of the amplified complementary DNA were run to assess the homogeneity of the PCR products and to test for the presence of primer-dimers. The primers used are shown in Supplemental Table 1. Data from real-time PCR studies were expressed relative to added luciferase, which is equivalent to expression per testis.

### Tubule permeability

Testes from iDTR mice were collected 7 days or 30 days after treatment with DTX at 50 days, and tubule permeability was tested *in vitro* as described previously (8).

### Hormone assay

Serum testosterone was measured using a commercial kit (Demeditec Diagnostics GmbH, Kiel, Germany), and serum LH was measured by enzyme-linked immunosorbent assay as previously described (24). The interassay coefficient of variation (CV) for testosterone was 10.9% at 2.5 ng/mL and 8.9% at 6.0 ng/mL, whereas the intra-assay CV was <8% for 0.1 to 25 ng/mL. The interassay CV for LH was 12% at 1.2 and 2.5 ng/mL, whereas the intra-assay CV was <10% for 0.1 to 10 ng/mL.

### Statistics

Data sets were analyzed using general linear modeling. When this showed a significant overall difference, the significance of difference between individual groups was determined by *t* tests using the pooled variance. When appropriate, data were logged to avoid heterogeneity of variance. Initial correlations between data sets were analyzed using the Pearson correlation coefficient except when data were visually assessed to differ markedly from a bivariate normal distribution; in that case, Spearman rank correlation was used instead, with *P* value calculated by a randomization test. Discriminant analysis was also used to analyze germ cell data. Analysis was carried out using Minitab 15 (Minitab Ltd, Coventry, UK) or Graph Prism version 5 (GraphPad Software Inc., San Diego, CA). No differences were observed between adult control iDTR animals treated with vehicle as neonates and those treated as adults, and results from these animals were combined when appropriate. Sertoli cell and Leydig cell numbers in adult controls from DTA and iDTR mice were significantly different and were treated separately for analysis. This difference probably arises from differences in the background of the two colonies.

To predict adult testicular cell composition, specifically Leydig cell counts and germ cell counts, we evaluated a range of linear models fitted to the data (DTX-treated mice). Candidate models included terms for Sertoli cell count and additional terms for the square root and/or the square of the count. We assessed age as a continuous predictor and as a categorical variable. We considered age as a category because we expected a discontinuous age effect. In total, three age categories were used: prepubertal (<21 days); pubertal (21 to 60 days for Leydig cell prediction and 21 to 45 days for germ cell prediction), and adult (>60 and >45 days for Leydig cell prediction and germ cell prediction, respectively). Each age category included all animals euthanized at that age, irrespective of when Sertoli cell ablation occurred. We also tested first-order interactions between the Sertoli cell counts (and their squares and square roots) and the age category to allow coefficients for these variables to vary by age group. Models were selected to minimize small sample-corrected Akaike Information Criteria (23), a parameter count penalized measure of model fit, aiming to identify the best predictive model and reduce the risk of overfitting. Linear modeling assumptions were assessed by graphical examination of model residuals for approximate normality and heteroscedasticity, with the provision to log transform dependent variables (Sertoli cell count and germ cell count) when residuals were heteroscedastic. Best-fitting models of Leydig cell and germ cell counts are presented as estimated parameters and formulae for each mouse age class and graphically plotted against the Sertoli cell counts by age group. We assessed the models’ predictive performance using leave-one-out cross-validation—sequentially holding back data from one mouse at a time, reestimating the best model on the remaining data, and using the resulting model to predict the cell count for the held-back animal. The R statistical system (version 3.3.1; 2016-06-21) (25) was used for the development of the linear models, the analysis of their results (packages AICcmodavg and tidyverse), and randomization tests for Spearman correlation coefficients (coin package).

## Results

### Sertoli cell population size is reduced in DTA mice and can be selectively reduced in iDTR mice by DTX injection in a dose-dependent manner

**Figure 1.**
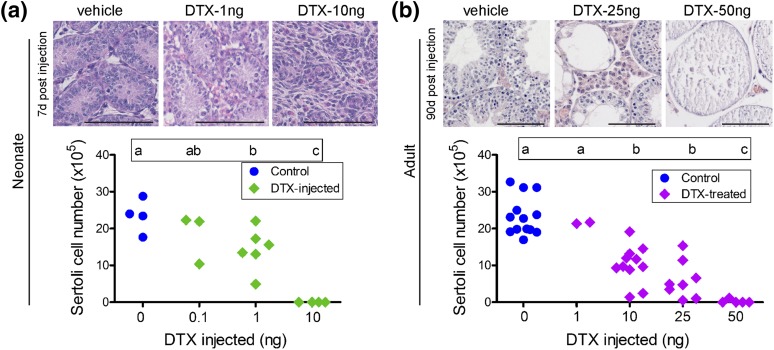
Changes in Sertoli cell number after treatment with different doses of DTX. (a) Neonatal (2 days) or (b) adult (50 days) iDTR mice were injected subcutaneously with different doses of DTX, and Sertoli cell numbers were measured when the animals were (a) 9 days old (7 days after injection) or (b) 80 days old (30 days after injection). Sertoli cell numbers for individual animals are shown. Differences between groups are shown by the letters above each group; groups that do not share a common letter are significantly different. Examples of hematoxylin and eosin−stained sections from different groups of animals treated with DTX (a) as neonates or (b) as adults are shown above each graph. The bars represent 100 µm.

To assess the impact of defined reductions in Sertoli cell numbers on testis development and function, we first sought to establish predictable models correlating exposure to DTX to Sertoli cell number. Because DTX does not cross the placenta, for prenatal reductions in Sertoli cell number we again exploited the Sertoli cell-specific Amh-Cre:DTA (DTA) mouse line, in which expression of DTA is controlled by Cre-mediated induction of DTA gene expression, leading to Sertoli cell death. We previously reported that a proportion of DTA mice showed only partial ablation of the Sertoli cell population *in utero* (9), and these animals were now suitable for our study of the effects of reduced Sertoli cell number in fetal life. To determine whether it is possible to induce partial ablation of the Sertoli cell population in the neonate or in adulthood in a controlled fashion, the effects of different doses of DTX were tested using Amh-Cre:DTR (iDTR) mice (which exclusively express the receptor for DTX in Sertoli cells) (Fig. 1). In neonatal mice (aged 2 days), treatment with 1 ng DTX caused a variable ∼50% reduction in Sertoli cell numbers when examined on day 9, whereas 10 ng caused complete Sertoli cell ablation [Fig. 1(a)]. Preliminary studies indicated that subcutaneous injection of adult mice with 1 ng DTX had no effect on Sertoli cell number [Fig. 1(b)], but more detailed studies showed that both 10 and 25 ng caused a clear, variable (mean, ∼50%) reduction in cell numbers **(**apparent 7 days after injection [Supplemental Fig. 2(a)]**)**, whereas injection of 50 ng DTX caused complete Sertoli cell ablation [Fig. 1(b)]. After partial ablation of the Sertoli cell population in the neonate, testis volume did not differ from that of controls until day 80 [Supplemental Fig. 2(b)]. Reducing Sertoli cell numbers in the adult reduced testis volume in parallel with changes in Sertoli cell number after 7 days [Supplemental Fig. 2(c)]. For subsequent studies, neonatal animals were treated with 1 ng DTX and adults were treated with 10 or 25 ng DTX to induce a partial reduction in Sertoli cell numbers. The variable response between animals means that in most cases, data from individual animals are shown in the following studies.

### Sertoli cell number does not recover after partial ablation during development or in adulthood

**Figure 2.**
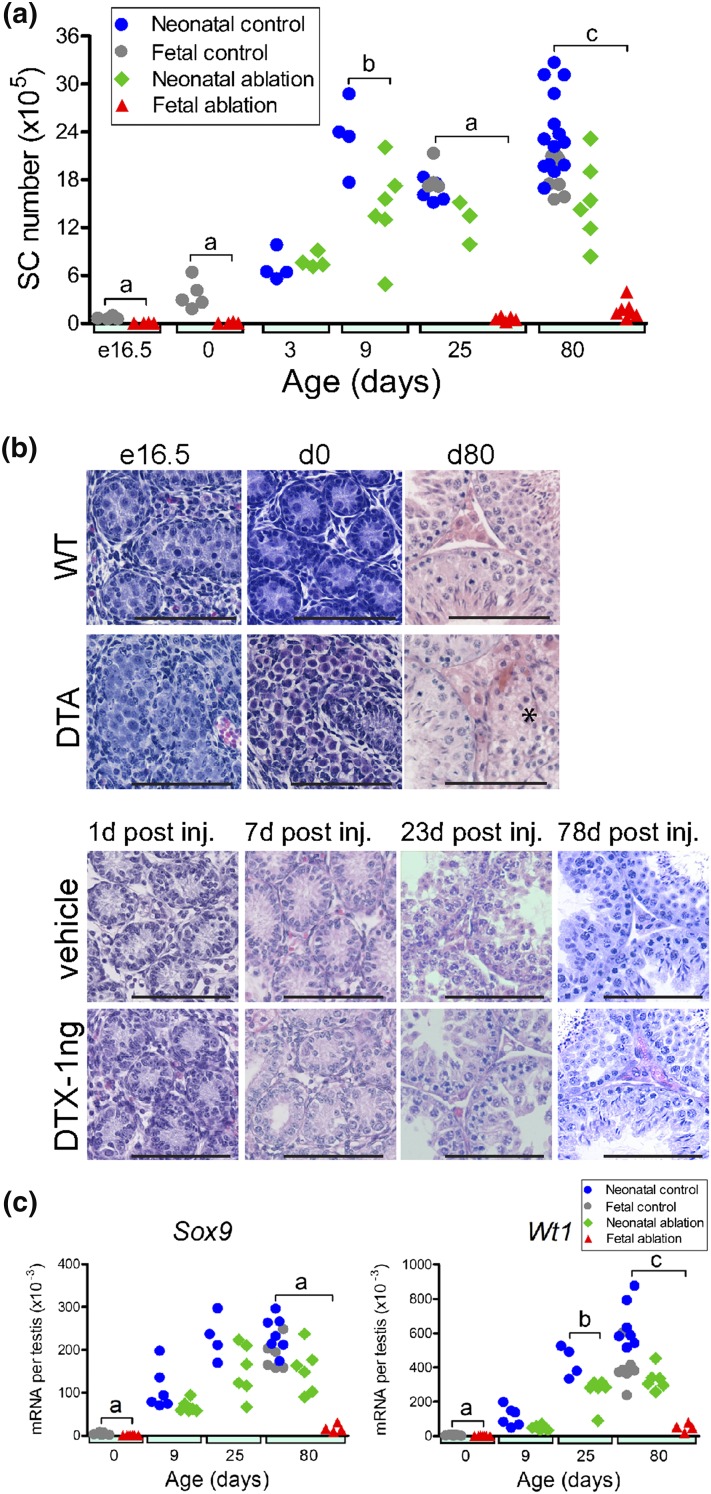
Effects of Sertoli cell ablation on Sertoli cell number, morphology, and transcript levels in the fetus and neonate. (a) Combined data from studies on fetal and neonatal Sertoli cell ablation show changes in Sertoli cell number in each group from e16.5 to adulthood. (b) Testis morphology during development in control mice (WT), DTA mice, and iDTR mice treated as neonates (2 days) with 1 ng DTX or with vehicle. Sections are shown from animals at ages e16.5, birth (day 0), and 80 days (WT and DTA mice) or at 3 days [1 day after injection (inj)], 9 days (7 days after injection), 25 days (23 days after injection), and 80 days (78 days after injection) (iDTX mice). (c) Sertoli cell transcript levels (expressed per testis) in control animals and after Sertoli cell ablation in the fetus or the neonate; data from individual animals are shown. Cell numbers and transcript levels in control animals used for fetal (DTA mice) or neonatal (iDTR mice) ablation were significantly different, and control groups were not combined for statistical analysis. When significant differences between groups occurred at each age, these differences are indicated by a bar and letter at each age: a, effect of fetal ablation was significant; b, effect of neonatal ablation was significant; c, effects of fetal ablation and neonatal ablation were both significant. No bar is shown when there was no significant difference between groups at that age. Numbers of Sertoli cells in the DTA group increased significantly (*P* < 0.05) from birth to 25 days and from 25 days to adulthood. (a and b) The bars represents 100 µm. (b) The asterisk in the adult DTA mouse section shows a seminiferous tubule lacking normal spermatogenesis.

Sertoli cells undergo intense proliferation in fetal and early postnatal life (26, 27). To determine whether homeostatic mechanisms regulate Sertoli cell population size during this period to compensate for a reduction in cell number [as suggested (28)], we tracked the number of Sertoli cells in the testis after an induced reduction in either fetal (e16.5) or neonatal life (day 2). Sertoli cell numbers in control animals increased from e16.5 to reach their maximum at postnatal day 9 and remained at that level until adulthood [Fig. 2(a)]. In contrast, in DTA mice, which undergo partial ablation of the Sertoli cell population in fetal life, Sertoli cell number was significantly reduced at e16.5 to between 5% and 12% of that of controls [Fig. 2(a)]. Cross sections taken from the testes of these mice at e16.5 contained a reduced number of tubular sections (as low as one in some animals), indicating a reduced number of tubules [Fig. 2(b)]. Sertoli cell number did not increase between e16.5 and birth in DTA mice, although numbers did increase significantly from birth to day 25 and increased significantly again to adulthood (relative to day 25) [Fig. 2(a)]. However, Sertoli cell numbers in DTA mice were significantly reduced compared with those of control animals at all ages. In iDTR mice treated with 1 ng DTX on day 2 (partial neonatal Sertoli cell ablation), Sertoli cell numbers were normal on day 3 but were significantly reduced on day 9 and remained significantly reduced in adulthood [Fig. 2(a)], indicating little or no recovery in cell numbers during this period. Levels of the Sertoli cell−specific messenger RNA transcripts *Sox9* and *Wt1* reflected Sertoli cell numbers after fetal or neonatal ablation of the cells [Fig. 2(c)].

**Figure 3.**
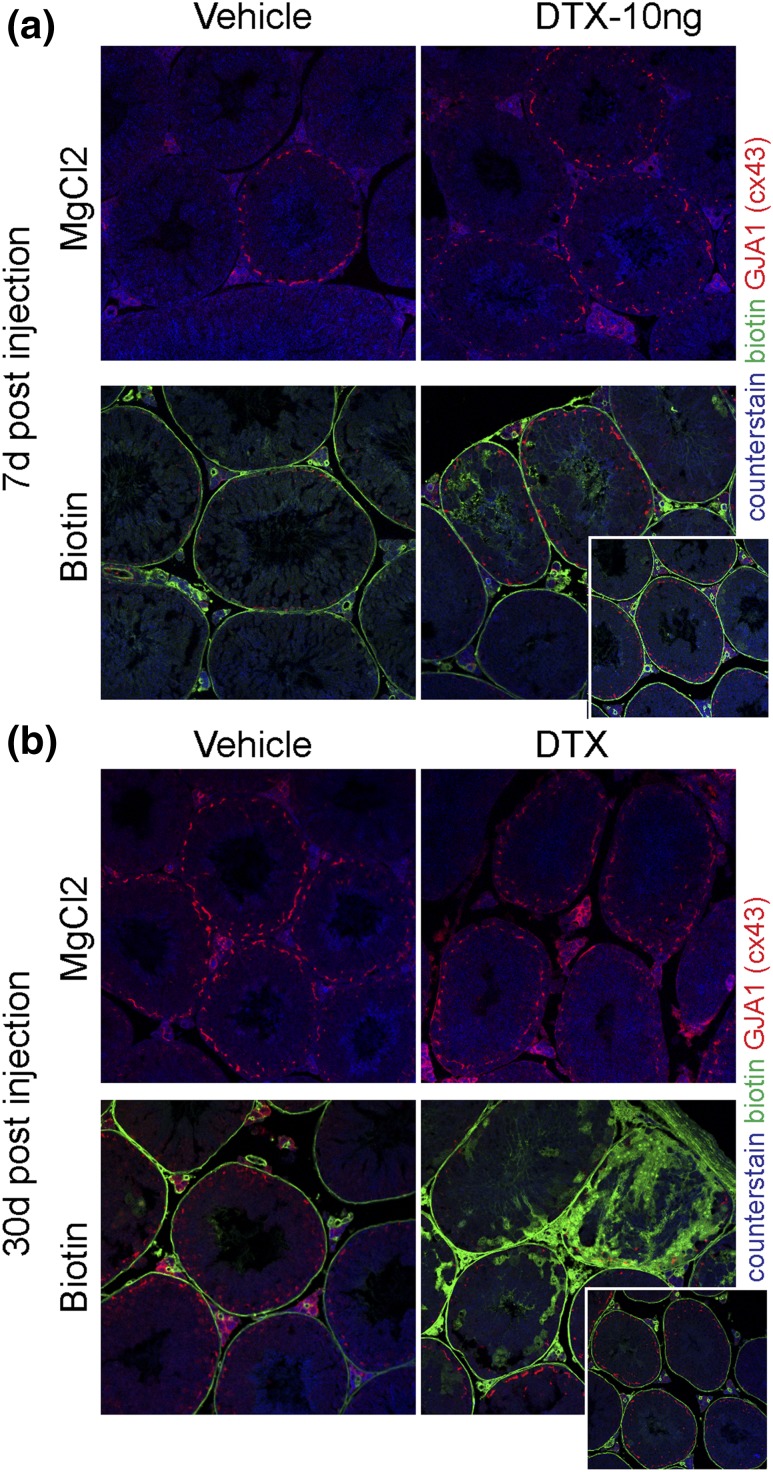
Effect of partial ablation of the Sertoli cell population on the BTB. To determine whether BTB integrity was maintained after partial ablation of the Sertoli cell population, adult (50 days) iDTR mice were treated with DTX (10 ng). Testes were injected with biotin or vehicle (solution containing MgCl_2_) *in vitro,* and biotin infiltration into the tubules was assessed (a) after 7 days or (b) after 30 days. Biotin infiltration into the lumen was seen in some tubules 7 days after DTX injection and, more markedly, 30 days after injection. However, most tubules were unaffected by partial ablation of the Sertoli cells (inserts at the bottom right of each figure), indicating that the remaining cells were able to establish a viable BTB.

To determine whether the continued increase in Sertoli cell number in DTA mice beyond 25 days was associated with retained expression of the immature Sertoli cell marker *Amh*, levels of *Amh* per Sertoli cell were measured in these mice (Supplemental Fig. 3). Results showed that adult *Amh* levels per Sertoli cell were not significantly different in DTA mice compared to control. Similarly, other markers of Sertoli cell development (*Sox9* and *Cst12*) did not differ between groups, although expression of *Rhox5* was significantly increased in DTA mice at birth and was reduced in adulthood (Supplemental Fig. 3). As expected, there was no recovery in Sertoli cell number for up to 90 days after partial ablation of Sertoli cells in adulthood [Supplemental Fig. 2(a)]. Loss of Sertoli cells by treatment of adult animals with DTX was accompanied by focal disruption to the blood−testis barrier (BTB), although most tubules appeared to maintain a viable BTB, suggesting that the remaining Sertoli cells are able to reestablish functional cell-cell junctions (Fig. 3).

Together, these data argue against the existence of a regulatory mechanism within the testis that changes Sertoli cell proliferation to rescue any shortfall in numbers.

### Total germ cell numbers and composition in adulthood are dependent on the number of Sertoli cells that form during development

Previous studies have demonstrated a correlation between Sertoli cell numbers and spermatid numbers (29–32), but it is unknown whether this applies across germ cell types. We took this opportunity to empirically examine the relationship between Sertoli cell population size and germ cell number in greater detail using our models, which are independent of gene knockout or endocrine modulation.

After partial ablation of the Sertoli cell population in the fetus, a proportion of the few tubules that remained in the adult had clearly disrupted spermatogenesis [Fig. 2(b), asterisk], although spermatogenesis in other tubules appeared largely normal. Partial ablation of the Sertoli cell population in the neonate reduced adult tubule diameter, but ongoing spermatogenesis was apparent in most tubules [Fig. 2(b)] with qualitatively normal spermatogenesis and an absence of germ cell−free tubules.

**Figure 4.**
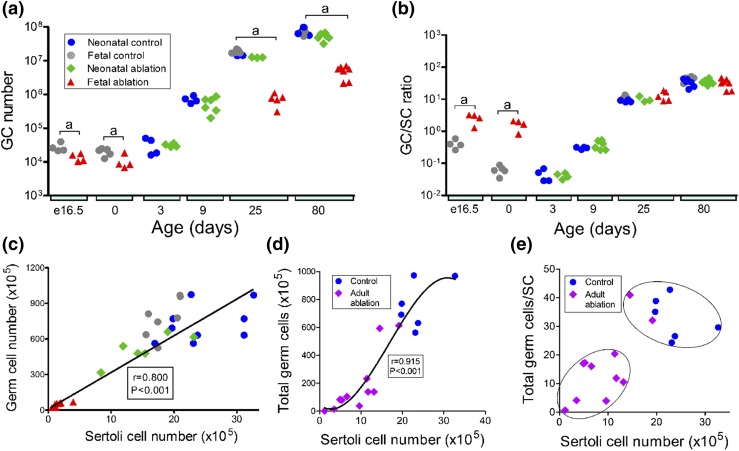
Effects of Sertoli cell ablation on germ cell (GC) number and germ cell/Sertoli cell (GC/SC) ratio in the fetus or neonate. (a and b) Combined data from studies on fetal and neonatal Sertoli cell ablation showing changes in total GC number and GC/SC ratio in each group from e16.5 to adulthood. Data from individual animals are shown. Cell numbers in control animals used for fetal or neonatal ablation were significantly different, and control groups were therefore not combined for statistical analysis. When significant differences between groups occurred at each age, these differences are indicated by a bar and letter at each age: a, effect of fetal ablation was significant; b, effect of neonatal ablation was significant; c, effects of fetal ablation and neonatal ablation were both significant. No bar is shown when there was no significant difference between groups. Note the use of a logarithmic scale for data on GC number and GC/SC ratio. (c) Correlation between total GC number and Sertoli cell number in adult control mice, DTA mice, and iDTR mice treated with DTX as neonates (Spearman *r* = 0.800; *P* < 0.001; when the fetal ablation group was excluded from the analysis, *r* = 0.543; *P* = 0.013). (d and e) Changes in total GC number and GC/SC ratio after Sertoli cell ablation in the adult. (d) There was a significant correlation (Pearson correlation coefficient, *r* = 0.915; *P* < 0.001) between total GC number and Sertoli cell number, and the best fit line was a second-order polynomial with the equation *Y* = 38.68*X* − 0.0716*X*^2^ − 142.4. (e) GC/SC ratios were assessed by discriminant analysis, and identified groups have been circled where appropriate.

Total numbers of germ cells were static from e16.5 to birth and then increased continuously to adulthood in control animals [Fig. 4(a)]. Gonocyte number was significantly reduced at e16.5 after partial ablation of the Sertoli cell population in fetal life and did not change significantly up to birth [Fig. 4(a)]. Gonocytes were seen only in the tubules of control mice but were present in both the tubules and the interstitium of the testes at e16.5 and at birth in DTA mice (Supplemental Fig. 4). After birth, there was a marked increase in germ cell numbers up to day 80 in DTA mice, though this remained significantly less than numbers of germ cells in control animals [note log scale in Fig. 4(a)]. After an induced reduction in Sertoli cell numbers in the neonate, mean germ cell numbers were not significantly different from those of controls at any later age.

The germ cell/Sertoli cell ratio (an indication of the number of germ cells supported by each Sertoli cell) decreased in control animals between e16.5 and day 0 (because of the relatively greater increase in Sertoli cell number between these ages). The ratio then increased after day 3, reaching a maximum at day 80 [Fig. 4(b)]. After partial ablation of the Sertoli cell population at e16.5, the germ cell/Sertoli cell ratio was significantly greater than that of control animals at e16.5 and day 0 but was the same as that of controls on days 25 and 80. The germ cell/Sertoli cell ratio was normal at all ages after neonatal ablation of Sertoli cells [Fig. 4(b)].

Analysis of all data points from all groups revealed a significant correlation (*r* = 0.800; *P* < 0.001) between total germ cell number in adulthood and size of the Sertoli cell population that formed during development [Fig. 4(c)], demonstrating that Sertoli cell number is a predictable biomarker of total germ cell number under nonpathological conditions.

### A marked reduction in Sertoli cell numbers in the adult is required to alter the germ cell/Sertoli cell ratio

Partial ablation of the Sertoli cell population at day 50 reduced tubule diameter 30 days later, but ongoing spermatogenesis was apparent in most tubules [Fig. 1(b)]. However, this spermatogenesis appeared abnormal, and it was not possible to categorize normal spermatogenic stages in most tubules. Most animals contained some tubules that were largely germ cell free [Fig. 1(b), 25 ng], and the numbers of these tubules increased in animals with greater levels of Sertoli cell ablation.

Total germ cell numbers were again closely correlated to Sertoli cell numbers after an induced reduction of Sertoli cell numbers in the adult [Fig. 4(d)] (*r* = 0.915; *P* < 0.001). This relationship was also apparent when individual germ cell types (spermatogonia, spermatocytes, round spermatids, and elongated spermatids) were plotted against Sertoli cell numbers [Supplemental Fig. 5(a)]. Similarly, the total germ cell/Sertoli cell ratio was dependent on Sertoli cell number, but discriminant analysis showed the presence of two distinct groupings [Fig. 4(e)]. These two groupings were also present when total germ cell numbers were subdivided into spermatocytes, round spermatids, and elongated spermatids [Supplemental Fig. 5(b)]. In these germ cell types, the germ cell/Sertoli cell ratio remained normal with declining Sertoli cell numbers until Sertoli cell numbers decreased below 60% to 65% of normal. Below this level, the germ cell/Sertoli cell ratio declined toward zero as Sertoli cell numbers decreased. Spermatogonial cell number per Sertoli cell appeared to show a different pattern of response to declining Sertoli cell number, with the ratio remaining normal or close to normal even when 80% of Sertoli cells were lost [Supplemental Fig. 5(b)].

### Leydig cell number is highly correlated with Sertoli cell number

**Figure 5.**
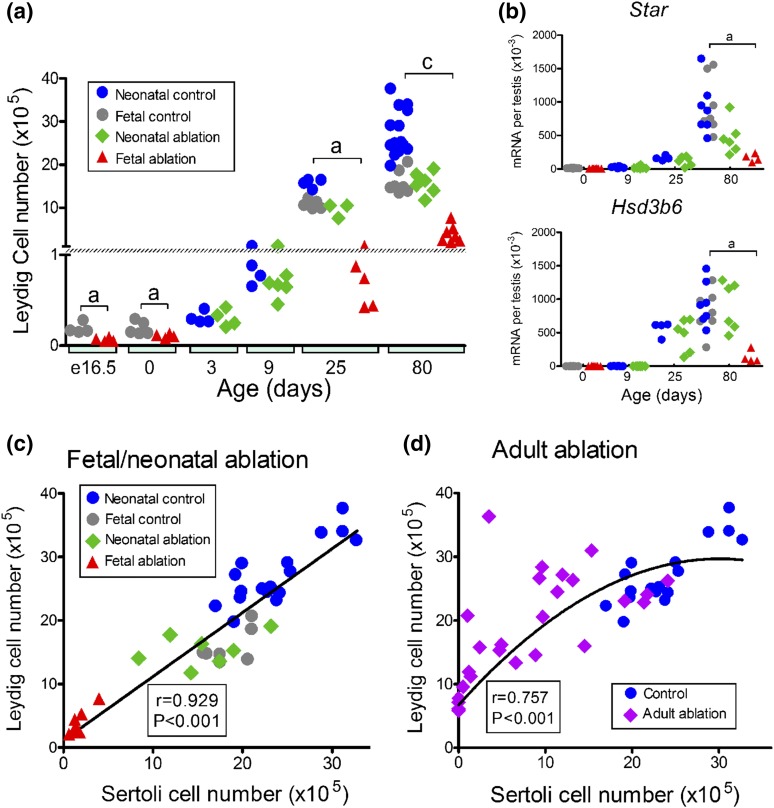
Changes in Leydig cell number after a reduction in Sertoli cell number during development or in the adult. (a) Combined data from studies on fetal and neonatal Sertoli cell ablation show changes in Leydig cell number during development. (b) Changes in Leydig cell transcript expression after Sertoli cell ablation in the fetus or neonate. (c) Correlation between Leydig cell number and Sertoli cell number in the adult after fetal or neonatal loss of Sertoli cells (*r* = 0.929; *P* < 0.001). (d) Correlation between Leydig cell number and Sertoli cell number after adult loss of Sertoli cells. The line drawn indicates the best fit constrained to (*X* = 0, *Y* = 6.725), which is the mean of the consistent Leydig cell numbers seen in the absence of Sertoli cells. The curve represents a second-order polynomial best fit with *Y* = 6.725 + 1.524*X* − 0.0253*X*^2^ (Pearson correlation coefficient, *r* = 0.757; *P* < 0.001). Data from individual animals are shown. Cell numbers and transcript levels in control animals used for fetal or neonatal ablation were significantly different, and control groups were therefore not combined for statistical analysis. When significant differences between groups occurred at each age, these differences are indicated by a bar and letter at each age: a, effect of fetal ablation was significant and c, effects of fetal ablation and neonatal ablation were both significant. No bar is shown when there was no significant difference between groups. mRNA, messenger RNA.

During development, Leydig cell numbers did not change significantly in control animals between e16.5 and day 3 but then increased through puberty reaching a maximum by day 80 [Fig. 5(a)]. Partial ablation of the Sertoli cell population in fetal life (DTA mice) reduced Leydig cell numbers at all ages up to day 80 [Fig. 5(a)]. Partial neonatal ablation of the Sertoli cell population had no significant effect on Leydig cell numbers up to day 25, but it significantly reduced Leydig cell numbers in adulthood [Fig. 5(a)]. Transcript expression of the Leydig cell markers *Star* and *Hsd3b6* largely paralleled changes in Leydig cell numbers [Fig. 5(b)], although there was no difference in transcript levels between control animals and animals treated with DTX as neonates (neonate ablation).

When data from fetal and neonatal Sertoli cell ablation studies were combined, there was a very strong correlation (*r* = 0.929; *P* < 0.001) between numbers of Sertoli and Leydig cells in adulthood [Fig. 5(c)]. This means that the number of Leydig cells in the adult testis could be clearly predicted from the number of Sertoli cells that formed during development. A clear correlation (*r* = 0.757; *P* < 0.001) was also evident between numbers of Sertoli cells and Leydig cells when an induced reduction in Sertoli cell number was carried out in the adult [Fig. 5(d)].

**Figure 6.**
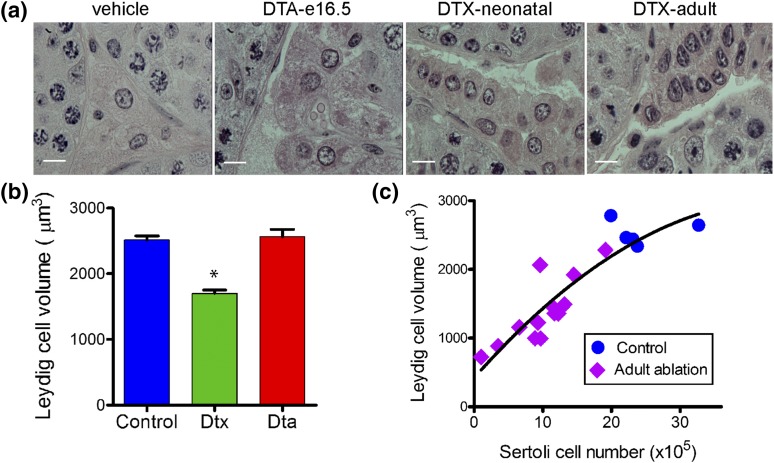
Changes in Leydig cell morphology and volume after Sertoli cell ablation. (a) Adult Leydig cell morphology in control animals and after partial ablation of Sertoli cells in the fetus (DTA mice), neonate (iDTR mice treated with DTX), and adult mice (iDTR mice treated with DTX). Leydig cell morphology was largely unchanged after fetal Sertoli cell ablation, but there was an apparent decrease in cell volume after neonatal or adult ablation of Sertoli cells. The bars in these images represent 10 µm. (b) Adult Leydig cell volume measured by the uniform isotropic random rotator method after Sertoli cell ablation in the fetus (DTA) or neonate (DTX). Data show mean ± standard error of the mean (n = 6 to 11 animals in each group). *P < 0.05 vs control. (c) Correlation between Leydig cell volume and Sertoli cell number in individual animals after adult Sertoli cell ablation (*r* = 0.891; *P* < 0.001).

Leydig cell morphology in the adult was normal after fetal ablation of Sertoli cells, but cell volume appeared reduced after neonatal or adult Sertoli cell ablation [Fig. 6(a)]. This was confirmed by stereological analysis [Fig. 6(b)], which also showed a strong correlation (*r* = 0.891; *P* < 0.001) between Sertoli cell number and Leydig cell volume after a reduction in Sertoli cell numbers in the adult [Fig. 6(c)].

No changes in LH or testosterone levels (Supplemental Fig. 6) were seen after partial ablation of the Sertoli cell population in the adult, which is consistent with previous observations from total Sertoli cell ablation studies (9).

### Prediction of testicular cell composition using linear models

Given the strong correlation between Sertoli cell number and both Leydig cell and germ cell numbers, we considered the possibility of developing linear models to predict adult testis cell composition from the number of Sertoli cells. For both Leydig cell count and germ cell count, model residuals demonstrated a marked increase in variance with increasing predicted values, so further analysis was performed after log transformation of dependent variables (Leydig and germ cell counts) (see Supplemental Fig. 7 for model residual plots). Model fit for key models as assessed by Akaike Information Criteria is shown in Supplemental Table 2. The best linear model of (log) Leydig cell counts included age as a category and square root, linear, and squared terms for Sertoli cell counts (adjusted *r*^2^= 0.97). The model had interactions between age and Sertoli cell number so is most clearly represented as separate submodels for (log-transformed) Leydig cell count in each age category (see Supplemental Table 3a).

**Figure 7.**
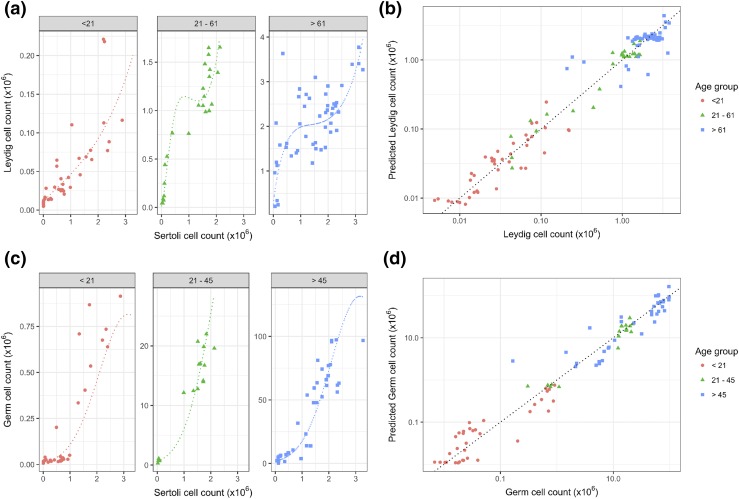
Development of linear models with which to predict testicular cell composition from Sertoli cell numbers. (a) Data for predicting Leydig cell number (counts) from Sertoli cell number in three age categories (<21, 21−61, and >61 days old). Dotted lines show fitted linear models that are curved with inflection points because they were fitted to log-transformed Leydig cell counts and include square root and linear and square terms. (b) Actual Leydig cell number plotted against predicted number from linear models after leave-one-out cross-validation. Points are colored according to the age category, and the line is an identity line of slope 1.0 and intercept 0. (c) Data for predicting germ cell number from Sertoli cell number in three age categories (<21, 21−45, and >45 days old). Dotted lines show fitted linear models that are curved because they were fitted to log-transformed Leydig cell counts and include linear and square terms. (d) Actual germ cell number plotted against predicted number from linear models after leave-one-out cross-validation. Points are colored according to the age category, and the line is an identity line of slope 1.0 and intercept 0.

Figure 7(a) shows the data for each age category and the fitted relationship between Sertoli cell number and Leydig cell number. The leave-one-out cross-validated predictions for the data set are also shown in Fig. 7(b). The best model for (log-transformed) germ cell counts included age as a category and linear and squared terms for the Sertoli cell counts (adjusted *r*^2^ = 0.96). Although there was no interaction between age group and Sertoli cell counts, this model is also presented as two submodels in Supplemental Table 2 for clarity, and comparison with models for Leydig cell counts. Figure 7(c) shows the fitted relationship between Sertoli cell number and germ cell number for each age class. The cross-validated predictions on the data are shown in Fig. 7(d). The models show that Sertoli cell number at any age during development or in the adult may be used, together with the age class, as a likely strong predictor of both germ cell and Leydig cell numbers in the mouse testis.

## Discussion

Previous studies have shown that Sertoli cells are critical for testis differentiation, development, and adult function (8, 9, 33). They control formation of the seminiferous tubules, Leydig cell development, peritubular myoid cell function, the BTB, and germ cell survival/development. Most of these functions have been shown in mouse models through gene knockout or complete cell ablation studies and so have only limited relevance to common, real-life problems such as developmental dysfunction or aging. Dysregulation of development (*e.g.,* through exposure to xenotoxins) or the effects of aging most likely cause only partial changes in cell number (34, 35); thus, it is important that we know how such changes can impact subsequent testis function. In this study, we show that partial loss of the Sertoli cell population during development or in the adult has significant knock-on effects, changing the Leydig cell number, the germ cell number, the BTB, and spermatogenesis. The study also identifies a fundamental relationship between Sertoli cell number, Leydig cell number, and total germ cell number, which most likely underpins the development and maintenance of the adult testis.

The most obvious effect of reducing the size of the fetal Sertoli cell population was a marked reduction in the number of testis cords, which was likely due to loss of basal lamina components secreted by the Sertoli cells (36). It was reported previously that Sertoli cell numbers can recover to normal levels by puberty despite an induced reduction in proliferation *in utero* (28, 37). However, the loss of testis cords in the DTA model of Sertoli cell ablation likely restricts the number of Sertoli cells, which can subsequently develop during the normal fetal and neonatal proliferative phases (27, 38), and provides one explanation of why there is no postnatal catch-up of Sertoli cell numbers.

There was, nevertheless, continued proliferation of Sertoli cells in these mice beyond the normal period of mitotic arrest at puberty. This may be related to altered endocrinology and/or delayed Sertoli cell maturation, although by day 80 levels of *Amh* (a marker of Sertoli cell immaturity) were not significantly different from those of control animals. No catch-up of the Sertoli cell population and no prolonged proliferation past puberty was seen in mice after an induced reduction in neonatal Sertoli cell numbers. This means that the Sertoli cell population is not able to adjust to late-onset dysregulation, a result consistent with that of a previous study showing that Sertoli cell numbers do not recover when postnatal proliferation is altered (34).

Numerous studies have shown that it is possible to increase Sertoli cell proliferation in the neonatal period [*e.g.,* through hemicastration, endocrine manipulation, or gene knockout (*e.g.,* 30, 39, 40)], but increased proliferation does not appear to occur when the population size is simply reduced after birth. In humans the late stage of Sertoli cell proliferation occurs shortly before puberty (3), and the final size of the Sertoli cell population may be particularly sensitive to alterations during this period.

The direct relationship between spermatid and Sertoli cell number has been known since Orth *et al.* (29) showed that a reduction in Sertoli cell proliferation in the rat neonate causes a parallel change in Sertoli cell and spermatid numbers in the adult. Around the same time, it was also reported that a strong correlation exists between Sertoli cell number and spermatid number in adult rats (41), and it was subsequently shown that increasing Sertoli cell proliferation in the neonatal period of the rat or mouse increases spermatid number and maintains the spermatid/Sertoli cell ratio (30–32).

Studies here have extended this work to confirm that total germ cell number is also dependent on Sertoli cell numbers when the Sertoli cell number changes postpuberty and to formalize the relationship using linear modeling. In addition, this work shows that the number of germ cells maintained per Sertoli cell remains constant even when 60% of Sertoli cells are ablated. Germ cell development is dependent on the specialized environment of the seminiferous tubule, and maintenance of the germ cell/Sertoli cell ratio indicates that the Sertoli cells in the adult are able to reorganize after a significant reduction in numbers in such a way that maintains that environment. This is consistent with the maintenance of the BTB in most of the observed tubule sections. The spermatogonia/Sertoli cell ratio may be maintained with more marked loss of Sertoli cell numbers in the adult because they are not dependent on the specialized environment of the tubules.

Despite the relative maintenance of germ cell numbers, however, overall spermatogenesis lost its normal organization and cell associations after Sertoli cell loss in the adult, to the extent that staging of the tubules was not possible. This loss of organization did not prevent development and maturation of elongated spermatids, showing that a degree of flexibility exists in the process. Interestingly, spermatogenic staging and cell associations in the adult appeared normal when Sertoli cell ablation occurred in the neonate. This suggests that the remaining Sertoli cells are able to adapt during the first wave of spermatogenesis to achieve and maintain normal organization of germ cell development and the BTB in the adult.

During development in mammals, at least two populations of Leydig cells develop. Shortly after differentiation of the testes, a fetal population of Leydig cells forms and is responsible for masculinization of the fetus (42). Later, between postnatal days 7 and 10 in the mouse, an adult population of Leydig cells develops, which exists alongside the fetal population and maintains adult androgen levels (43–46). We showed previously through complete Sertoli cell ablation studies that development of the adult population of Leydig cells is dependent on Sertoli cells (9). Results described here now show that there is a strong correlation between the number of Leydig cells that develop in the adult and the size of the Sertoli cell population. Because the technique used to manipulate Sertoli cell numbers in this study does not have a direct effect on the Leydig cells, it is highly likely that this relationship is causative and implies that the size of the Sertoli population during development (both in the fetus and neonate) determines the number of Leydig cells in the adult.

This newly identified relationship is particularly relevant with respect to our understanding of the control of testis development and function in the adult, and evidence from previous studies supports the hypothesis. For example, both Sertoli cell and Leydig cell numbers are reduced in mice with induced androgen receptor expression, specifically in the Sertoli cells (47, 48). Similarly, in mice lacking insulin and insulinlike growth factor 1 receptors on the Sertoli cells, there is an apparent proportional decrease in both Sertoli cells and Leydig cells (49), whereas follicle-stimulating hormone receptor (FSHR) knockout mice show a reduction in both Sertoli cell and Leydig cell numbers (50, 51). Leydig cells and Sertoli cells both increase in nitric oxide synthase 2–null mice (32), although the nitric oxide synthase 2 deficiency is not specific to the Sertoli cells in these animals and could affect both cell types independently. More tangentially, a correlation exists between Sertoli cell and Leydig cell numbers in different boar breeds (52).

On the other hand, given that evidence supporting this relationship between Sertoli cell and Leydig cell numbers is strong, animal models that do not fit the hypothesis are of considerable interest because they may point toward other mechanisms that control Leydig cell numbers. It is clear, for example, that LH is the primary regulator of Leydig cell number around puberty because mice that lack LH or the LH receptor show a marked reduction in Leydig cell numbers (27, 53, 54). Similarly, a mouse model of Klinefelter syndrome shows an increase in Leydig cell numbers in the adult (55). It is not known what happens to Sertoli cell numbers in these animals, but there is a significant increase in LH that most likely explains the changes in Leydig cell numbers. In contrast, in FSH *β* subunit knockout mice, there is a reduction in Sertoli cell numbers in the adult but no change in Leydig cell numbers (50, 51). Because there is a parallel decrease in both Leydig cells and Sertoli cells in the FSHR knockout mouse (50, 51), this suggests that constitutive FSHR activity, which is most likely to be high in FSH *β* subunit knockout mice, may be essential for the interaction between Sertoli cells and Leydig cells. In another animal model, the Follistatinlike 3 (FSTL3) knockout mouse, an increase has been observed in Sertoli cell number in the adult with no change in Leydig cell number (40). Therefore, this may be indicative of a role for FSTL3 in Leydig cell development, which is supported by the observation that Leydig cell number appears to be increased in FSTL3-overexpressing mice (56).

In addition to enhancing our understanding of testis development, the relationship between Sertoli cell and Leydig cell numbers during development may be of some clinical relevance. Given that there does not appear to be catch-up growth of the Sertoli cell population and the critical periods of Sertoli cell proliferation in the human are *in utero* and prepuberty (3, 4), exposure to factors that might affect Sertoli cell proliferation during this period [*e.g.,* environmental chemicals (34) or fetal nutrient restriction (57)] is likely to have knock-on effects, reducing the adult Leydig cell population size. Conversely, treatments that increase Sertoli cell number or activity are likely to increase the size of the Leydig cell population. For example, studies of development in a primate model have shown that pubertal treatment with follicle-stimulating hormone (FSH) increases Leydig cell number (58). This effect is likely to be through an increase in Sertoli cell number and/or cell activity and highlights the potential importance of FSH in achieving optimal adult fertility in the adult male (59).

We reported previously that complete ablation of the adult Sertoli cell population leads to a 75% reduction in Leydig cell number (8). By using a partial ablation model, we now show that Leydig cell number in the adult is also directly correlated with Sertoli cell number. This effect is more variable than the developmental regulation discussed previously, and a reduction in Leydig cell number is clearly seen only when the Sertoli cell population is decreased by about 50% or more. Nevertheless, this relationship may be of importance, particularly during aging, when Sertoli cell numbers have been shown to decline in some species including humans (10–15). The effect of aging on Leydig cell numbers in humans is less certain; most studies report a reduction in Leydig cell number with age (15, 60–62), although a more recent study reports no change (13). This discrepancy may be because there is considerable variation in Leydig cell numbers between individuals and because subject numbers tend not to be large in these studies.

Data relating to aging in other species are limited; in the horse, there is no change in Sertoli or Leydig cell numbers with age (63), whereas in the rat, Sertoli cell numbers decline without a clear effect on Leydig cell numbers (64, 65). This latter observation may be an indication that something else is acting to maintain Leydig cell numbers in the rat, such as increased circulating FSH levels (64, 66). Few other studies are relevant to this relationship, although Leydig cell apoptosis is reported to occur after Sertoli cell loss in BCLW-deficient mice (67), whereas the seasonal increase in Leydig cell number in the horse is preceded by an increase in Sertoli cell number (68). A relationship between Sertoli and Leydig cell numbers would also explain what limits adult Leydig cell population size, which is consistent through normal adult life and which returns to exactly pretreatment levels in rats after complete ablation with ethane dimethane sulfonate (69, 70).

In this study, altered Sertoli cell number was also closely associated with changes in Leydig cell volume. This relationship has also been seen during aging in both rats and humans (61, 64, 65) and suggests a Sertoli cell−regulated change in cell function. Reduced cell volume is normally associated with reduced cell activity, although circulating testosterone levels are maintained in these animals; this would suggest an increase in Leydig cell activity as the total number of Leydig cells is reduced. This is consistent with the increase in *Cyp11a1* seen previously in Leydig cells after Sertoli cell ablation (9), but it leaves open the question of why cell volume declines. There are a number of possible explanations, such as direct loss of Sertoli cell stimulation or indirect effects through changes in testicular vasculature after Sertoli cell ablation (6). Interestingly, normal Leydig cell volume was seen after fetal Sertoli cell ablation, which indicates either that the cells are able to adapt to loss of the Sertoli cells or that vascular development at this stage is sufficiently flexible to ensure normal capillary development in the interstitial tissues. During aging, there is increased vascular dysfunction in the testis (71), so the change in Leydig cell volume during aging may also occur because of reduced vascularization brought about by loss of Sertoli cells. Leydig cell volume can also be regulated directly by circulating LH (72), although this is unlikely to explain aging effects, as LH levels are either unchanged during aging [rat (65)] or are increased [human (73)].

This study shows that dysregulation of fetal or neonatal testis development can have major knock-on effects on cell numbers in the adult testis and that Sertoli cell number cannot recover from dysregulation during the neonatal phase. Interestingly, however, there is enough flexibility during development of the testis to allow the establishment of qualitatively normal spermatogenesis in the adult despite significant loss of Sertoli cells during development. Results from this study also describe the fundamental relationship that exists between Sertoli cell, Leydig cell, and germ cell numbers during development and in the adult testis. This relationship can be circumvented by changes in hormone levels but, under normal circumstances, is most likely to regulate both Leydig and germ cell numbers in the adult and during aging. Given the critical role that the Leydig cells play in both fertility and adult health through secretion of testosterone (74), identification of the mechanism linking adult Sertoli cell and Leydig cell populations is of considerable importance in understanding testis development and adult function.

## Appendix

**Appendix.** **Antibody Table**

**Peptide/Protein Target**	**Antigen Sequence (if Known)**	**Name of Antibody**	**Manufacturer, Catalog No.**	**Lot Number**	**Species Raised in; Monoclonal or Polyclonal**	**Dilution Used**	**RRID**
GJA1		Connexin 43 (GJA1, cx43)	Thermo Fisher Scientific, 71-0700	500476A	Rabbit; polyclonal	1/250	AB_2533973
SCP3		SCP3	Abcam Ltd., Ab15093	GR95161-2	Rabbit; polyclonal	1/1600	AB_301639
DDX4		DDX4	Abcam Ltd., Ab13840	GR159873-2	Rabbit; polyclonal	1/400	AB_443012

Abbreviation: RRID, research resource identifier.
