# Supplementary material for: Sertoli Cell Number Defines and Predicts Germ and Leydig Cell Population Sizes in the Adult Mouse Testis
Source: Endocrinology. 2017 Jul 5;158(9):2955–69. doi: 10.1210/en.2017-00196 (PMC5659676; doi:10.1210/en.2017-00196)
Supplement: Supplementary file 1 [file en.2017-00196.st1.pdf]

Supplementary Table 1

Primers and antibodies used

(a) Primers

Gene	Forward primer	Reverse primer
Sox9	cgcggagctcagcaagactctg	cgcggagctcagcaagactctg
Wt1	gctccagctcagtgaaatggacagaa	ggccactccagatacacgccg
Star	cgtcggagctctctgcttggttc	tcgtccccgttctcctgctg
Hsd3b6	gctccagactgggactgctgacac	aatcctctggcccaaaaaccctc
Rhox5	aggttcgcccagcatcgactg	gccgcagccctcctgatctt
Amh	tcctacatctggctgaagtgatatggg	aggttctgtgtgccccgcag
Cst12	ggatgacgattttgcctacaagttcct	ttctctctcctggaccttcctgca

(b) Antibodies

**Primary Antibody (Ab1) name**	**References**	**Dilution AbI**	**Secondary antibody (AbII) conjugated**	**Dilution AbII**	**Detection system**
**GJA1 (connexion43)**	ThermoFisher Scientific<br>71-0700	1/250	Peroxidase	1/200	IF
**SCP3**	Abcam Ltd.<br>Ab15093	1/1600	Peroxidase	1/200	IF
**DDX4**	Abcam Ltd.<br>Ab13840	1/400	Peroxidase	1/200	IF
**For BTB assessment with biotin**			Strepatvidin alexa 488<br>(Molecular Probes Inc s-11223)	1/200	IF

*IF: immunofluorescence

Supplementary Table 2

**Models of Leydig Cell Counts**	**K (number of parameters)**	**AICc**	**ΔAICc**
Final model:<br>log(leydig) ~ age + sertoli$^{0.5}$: age + sertoli : age + sertoli^{2} : age	13	133.8	0
Next best model:<br>log(leydig) ~ age + sertoli$^{0.5}$: age + sertoli : age	10	141.3	7.52
Model only sertoli and age group terms:<br>log(leydig) ~ sertoli + age + sertoli:age	5	333.7	199.95

**Models of Germ Cell Counts**	**K (number of parameters)**	**AICc**	**ΔAICc**
Final model:<br>log(germ_cells) ~ sertoli + age + sertoli^{2}	6	185.7	0
Next best model:<br>log(germ_cells) ~ sertoli + age_gp_germ_cell + sertoli^{2} + sertoli$^{0.5}$	7	186.6	0.90
Next best model:<br>log(germ_cells) ~ sertoli + age_gp_germ_cell + sertoli:age_gp_germ_cell + sertoli^{2}	8	190.0	4.22
Model only sertoli and age group terms:<br>log(germ_cells) ~ sertoli + age + sertoli:age	5	202.3.7	16.6

Model fit for best models (as assessed by AICc) and also models with only linear terms of Sertoli cell count for prediction of Leydig cel counts and germ cell counts. 'age' signifies relevant categorical age group. 'sertoli:age' signifies the statistical interaction between sertoli cell count and age group. AICc is the small sample corrected Akaike Information Criteria, a complexity penalized measure of model fit (lower values signify a better fit). ΔAICc is the difference in AICc between a model and the best fitting model. For the prediction of germ cell counts the second best model had a similar fit (ΔAICc = 0.90) to the best model so is also shown. For both Leydig cell counts and germ cell counts models with additional Sertoli cell terms had a markedly better fit as assessed by AICc when compared with models with only the linear Sertoli cell term.

Supplementary Table 3

(a)

Age group	Term	Estimate	SE	P-value
< 21 days	intercept	-4.889	0.186	< 0.001
	Sertoli cell number $^{0.5}$	2.169	0.982	0.029
	Sertoli cell number	-0.411	1.079	0.704
	Sertoli cell number ^{2}	0.066	0.224	0.769
21 – 61 days	intercept	-5.214	0.516	< 0.001
	Sertoli cell number $^{0.5}$	14.203	2.498	< 0.001
	Sertoli cell number	-10.502	2.505	< 0.001
	Sertoli cell number ^{2}	1.637	0.548	0.004
> 61 days	intercept	-1.698	0.490	< 0.001
	Sertoli cell number $^{0.5}$	5.350	1.646	0.002
	Sertoli cell number	-3.385	1.300	0.011
	Sertoli cell number ^{2}	0.418	0.181	0.023

Model terms for the Leydig cell linear models (using cell counts x 10^6) for each age group with coefficient estimate and standard error and P-value of test against a null hypothesis that the coefficient = 0. Adjusted $R^2 = 0.97$

(b)

Age group	Term	Estimate	SE	P-value
< 21 days	intercept	-4.518	0.140	< 0.001
	Sertoli cell number	2.728	0.262	< 0.001
	Sertoli cell number ^{2}	-0.431	0.103	< 0.001
21 – 45 days	intercept	-0.496	0.207	0.0190
	Sertoli cell number	2.728	0.262	< 0.001
	Sertoli cell number ^{2}	-0.431	0.103	< 0.001
> 45 days	intercept	0.566	0.171	0.001
	Sertoli cell number	2.728	0.262	< 0.001
	Sertoli cell number ^{2}	-0.431	0.103	< 0.001

Model terms for the germ cell linear models (using cell counts x 10^6) for each age group with coefficient estimate and standard error and P-value of test against a null hypothesis that the coefficient = 0. Adjusted $R^2 = 0.96$

Note, for both A and B the models are predicting $\log_e$(counts) and so to convert to raw counts they need exponentiation.

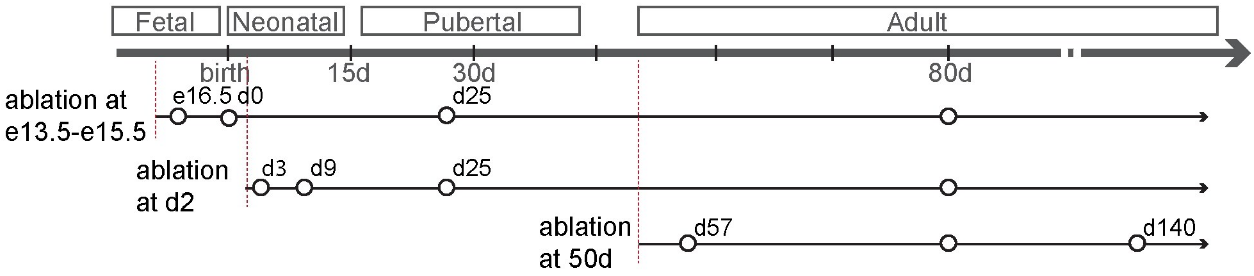

Supplementary Fig 1 Overview of experimental design. Schematic diagram indicating time points (e13.5 to e15.5, postnatal day 2 (d2) and postnatal day 50 (d50)) at which partial ablation of the Sertoli cell population occurred (vertical dotted lines). The timing of fetal ablation in DTA mice is not certain and is dependent on first expression of AMH-Cre. The circles indicate times at which animals were killed and tissues collected.

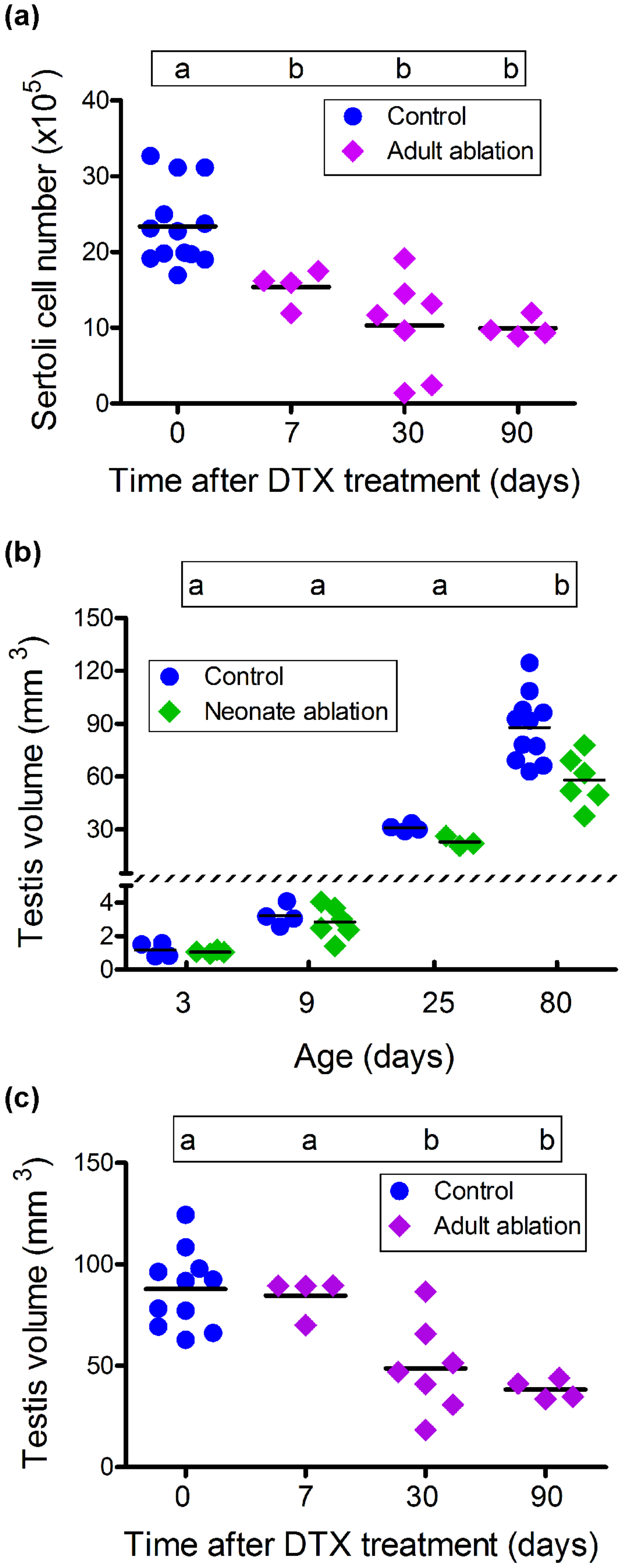

Supplementary Fig 2 Changes in Sertoli cell number and volume following partial ablation of the Sertoli cell population in the neonate or adult. (a) Changes in Sertoli cell number over time following treatment of adult (day 50) iDTR mice with 10ng DTX. (b) and (c) Changes in testis volume during development or over time following partial ablation of the Sertoli cell population in the neonate (day 2) (b) or adult (day 50) (c). Adults were injected with 10ng DTX and neonates were injected with 1ng DTX. In (a) and (c), differences between groups are shown by the letters above each group, groups which do not share a common letter are significantly different. In (b), ages at which there was no difference between control and DTX-treated animals are shown by an “a” while “b” indicates that there was a significant difference between treated and control animals. Data from individual animals is shown.

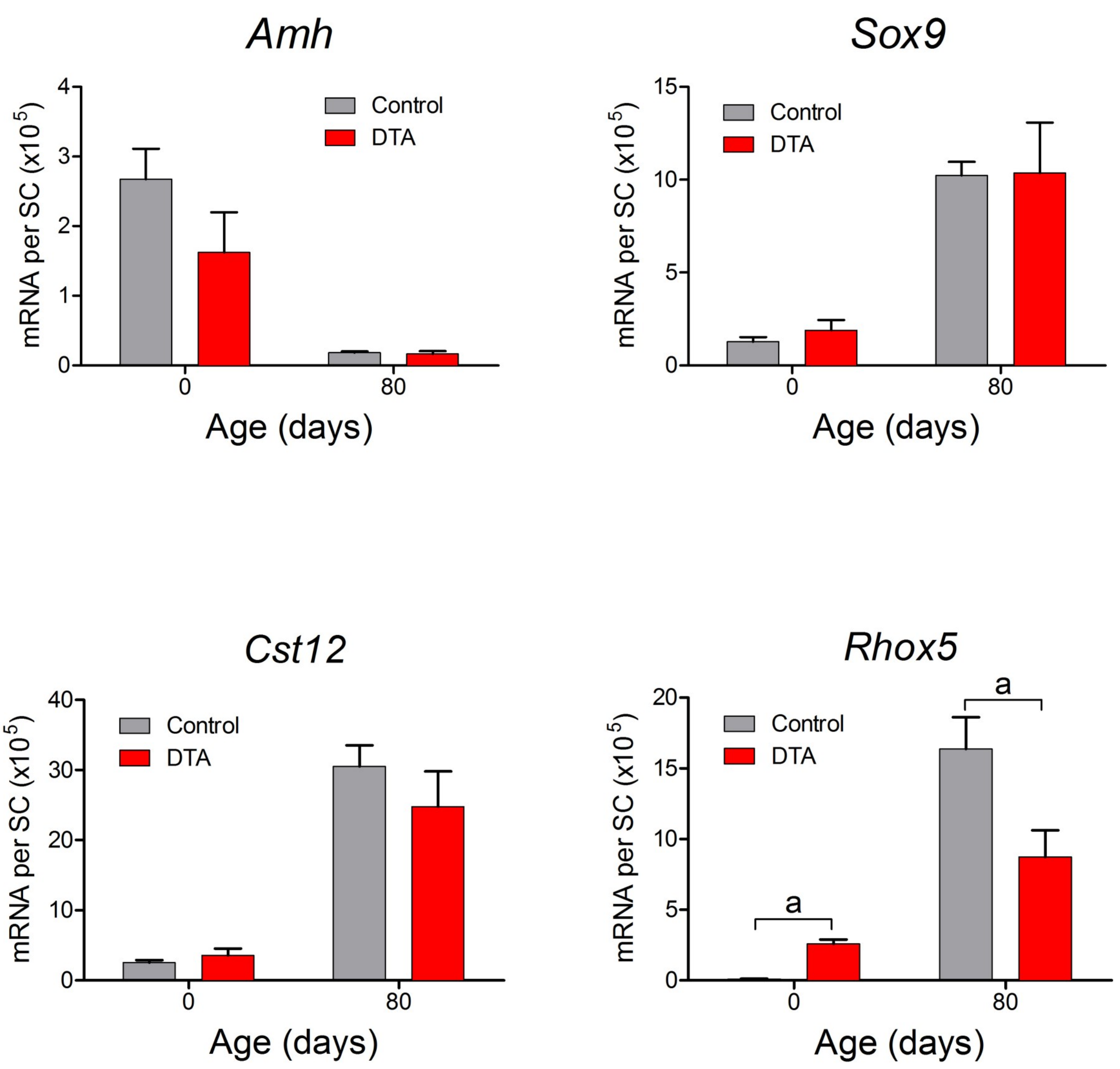

Supplementary Fig 3 mRNA transcript levels expressed per Sertoli cell in newborn and adult DTA mice and controls. Results show mean ± sem of 4-7 animals in each group. Differences between control and DTA mice were not significant for *Amh*, *Sox9* and *Cst12* but were significant at day 0 and day 80 for Rhox5 (as indicated by “a” on the graph) . The effects of age were significant in all 4 transcripts.

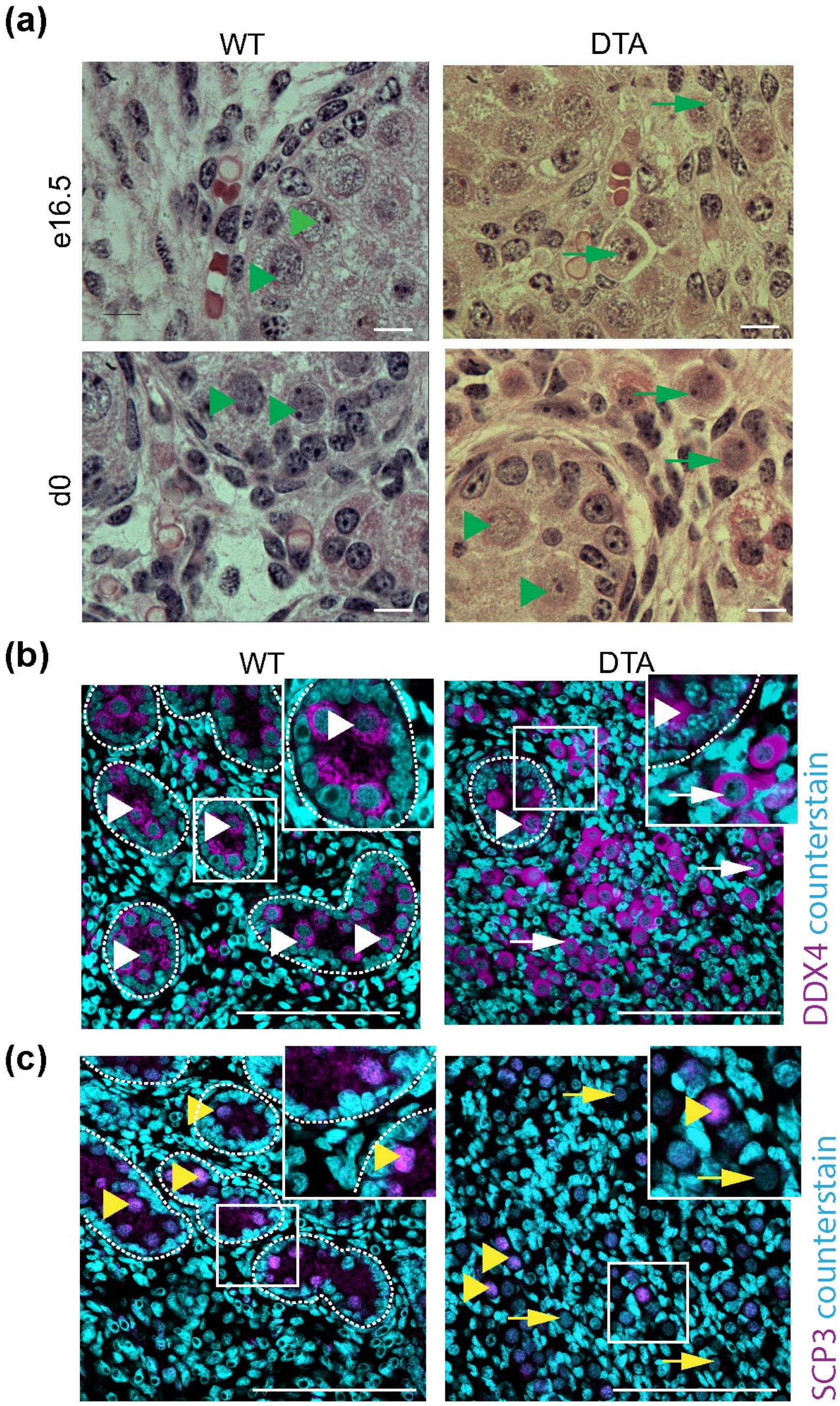

Supplementary Fig 4 Presence of gonocytes in the interstitium following partial ablation of the fetal Sertoli cell population. (a) Testis morphology at e16.5 and day 0 in control (WT) mice and following partial ablation of the Sertoli cell population in DTA mice. At e16.5 and d0 gonocytes are present only in the tubules (green arrowheads) of control animals. In contrast, in DTA mice gonocytes are present in both the tubules (green arrowheads) and in the interstitium (green arrows). (b) and (c) Immunohistochemical staining for the germ cell marker DDX4 and the germ cell differentiation marker SYCP3 on day 0. Tubules are outlined with a white dotted line and the boxed areas are shown enlarged in the top right corner of each image. Staining for DDX4 clearly shows the presence of interstitial gonocytes in DTA mice (white arrows) while only tubular gonocytes are present in control animals (arrowheads). In (c), some interstitial gonocytes in DTA mice had started to express the differentiation marker SYCP3 (yellow arrowheads) although most interstitial gonocytes did not express the protein (yellow arrows). In control mice some of the tubular gonocytes had started to express SYCP3. The bar represents 10μm in (a) and 500μm in (b) and (c).

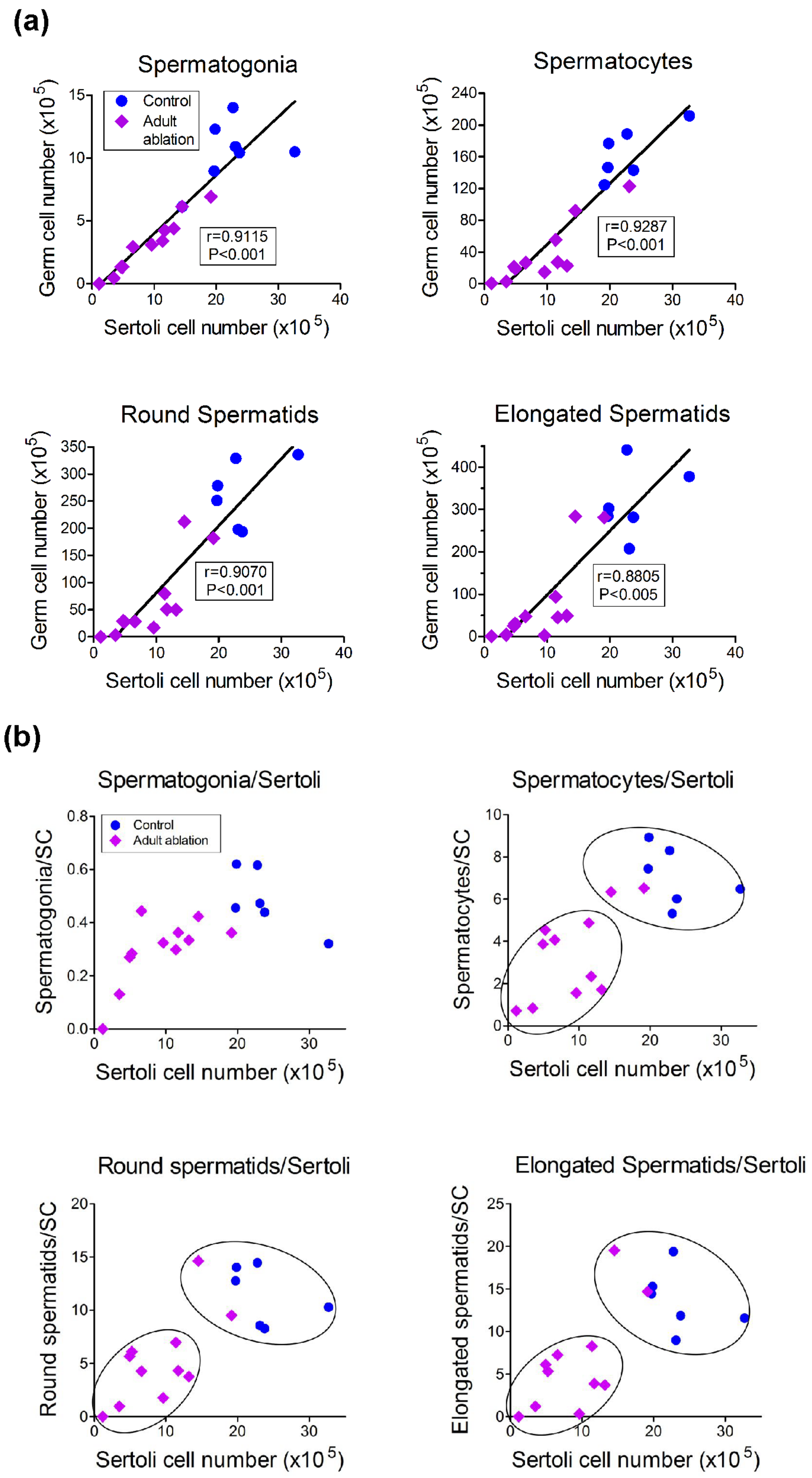

Supplementary Fig 5 Effect of partial ablation of the adult Sertoli cell population on numbers of specific germ cells types and on the ratio of specific germ cell types to Sertoli cells. Adult iDTR mice were treated with DTX to partially ablate the Sertoli cell population. Data shows (a) the numbers of each germ cell type and (b) the ratio of each germ cell types per Sertoli cell (SC) plotted against the number of Sertoli cells in each animal. In (a), Pearson's correlation co-efficients are shown for each germ cell type while in (b) the germ cell/Sertoli cell ratios were assessed by discriminant analysis and identified groups have been circled where appropriate.

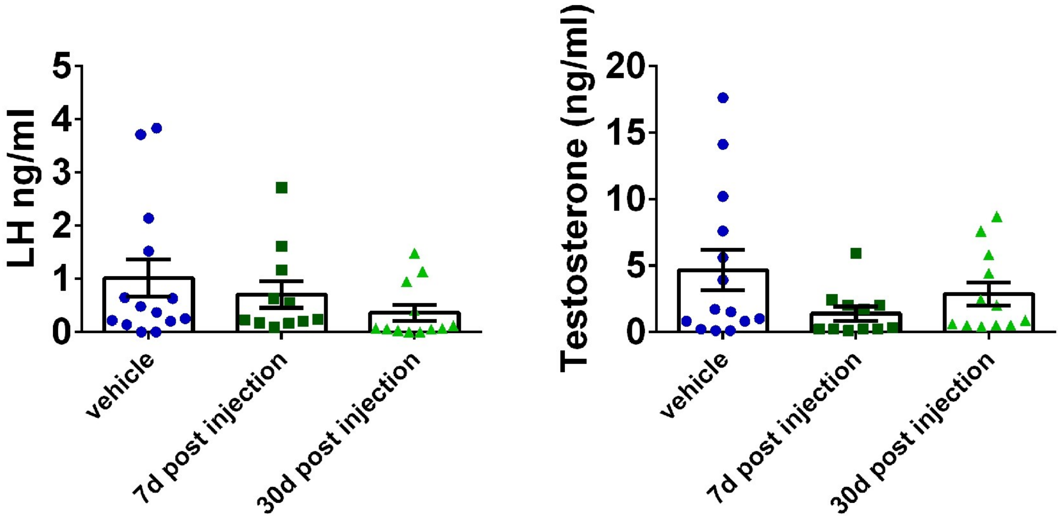

Supplementary Fig 6 Effect of partial ablation of the adult Sertoli cell population on plasma testosterone and luteinising hormone (LH) levels. Adult iDTR mice were treated with DTX to partially ablate the Sertoli cell population and hormone levels were measured 7d and 30d after injection. Data from individual animals is shown along with the mean ± SEM of each group. There were no significant differences between groups for either hormone.

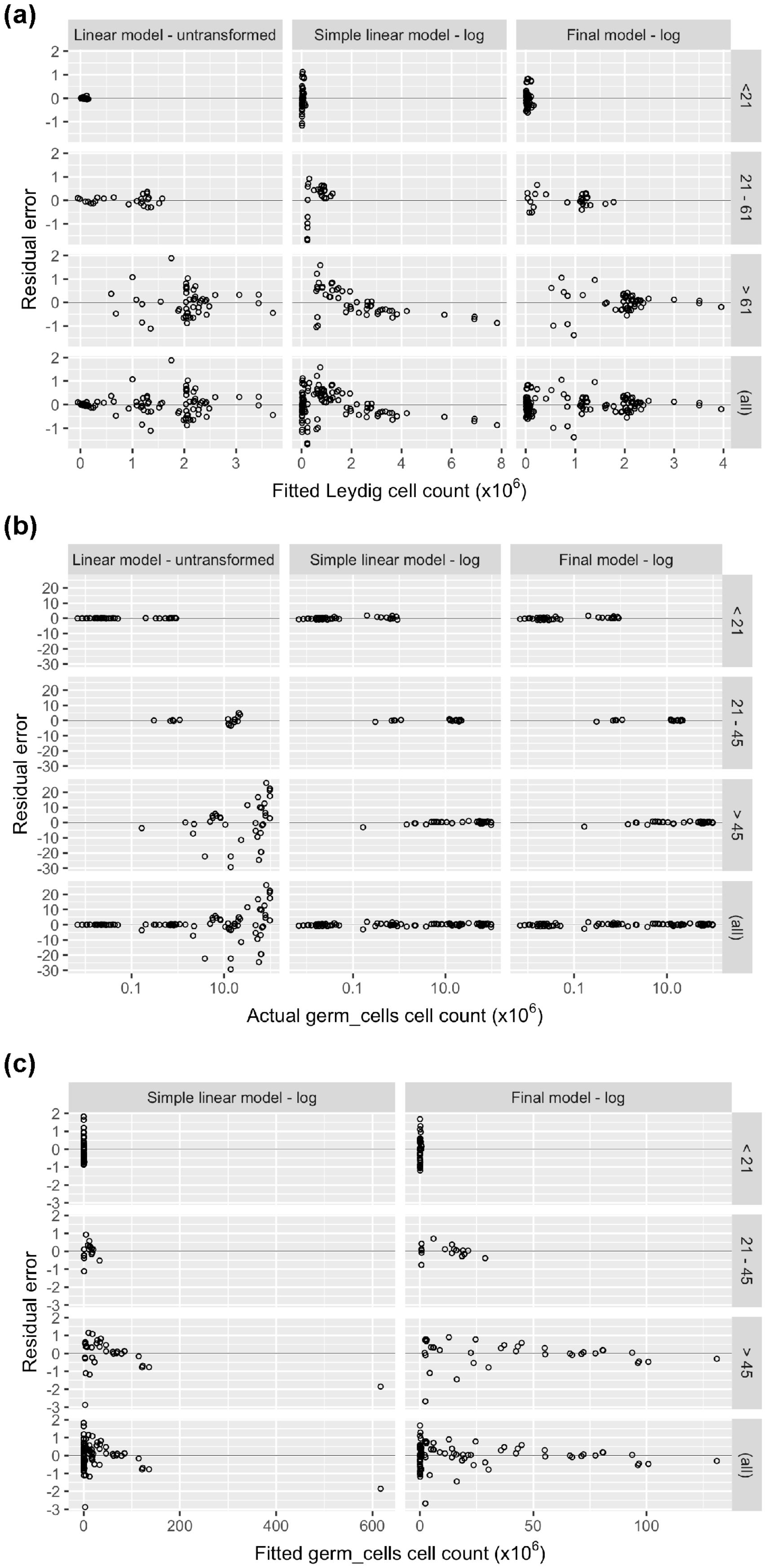

Supplementary Fig 7

(a) Model residuals plotted against predicted Leydig cell count values for a simple model using untransformed Leydig cell counts, a simple model using log$_e$ transformed counts and the final model which also included square root and squared terms for Sertoli cell count. The untransformed model plot demonstrates an increase in residual variance with increase in predicted Leydig cell count. The simple linear model with log$_e$ transformation plot shows a more stable variance structure but demonstrates a decreasing trend in residuals with increased Leydig cell count. The final model has a stable variance and absence of residual trend.

(b) Model residuals plotted against predicted germ cell cell count values for a simple model using untransformed germ cell cell counts, a simple model using log$_e$ transformed counts and the final model which also included squared terms for Sertoli cell count. The untransformed model demonstrates an increase in residual variance with increase in predicted germ cell cell count. The simple linear model with log$_e$ transformation and the final plot shows a more stable variance structure. The residual plots for log$_e$ transformed models are rescaled in (c)

(c) A decreasing trend in residuals present in the simple log$_e$ transformed model was partially removed by inclusion of the squared term for Sertoli cell counts in the final model. This model also had the best AICc assessed model fit.
